# Risk factors for drug resistance in allergen immunotherapy for allergic rhinitis: a systematic review and meta-analysis

**DOI:** 10.3389/falgy.2025.1743260

**Published:** 2026-01-23

**Authors:** Zhi-qiang Zhang, Jing-yang Li, Jinyu Geng, Xin-hua Zhu

**Affiliations:** 1 Department of Otorhinolaryngology, Head and Neck Surgery, The Second Affiliated Hospital, Jiangxi Medical College, Nanchang University, Nanchang, China; 2 Department of Clinical Medicine, The First Clinical Medical College, Nanchang University, Nanchang, China; 3 The First Clinical Medical College of Xinjiang Medical University, Urumqi, China

**Keywords:** allergen immunotherapy, allergic rhinitis, subcutaneous immunotherapy, sublingual immunotherapy, systematic review and meta-analysis

## Abstract

**Background:**

Allergen Immunotherapy (AIT) is largely considered to be the only therapy that can provide relief from allergic rhinitis (AR).Although its effectiveness has been confirmed by the results of a large number of practical studies such as randomized controlled trials, it may in some cases have a poor or no response to treatment due to the development of resistance under the influence of certain risk factors. The purpose of this Meta-analysis was to examine the risk factors for AR resistance to AIT treatment.

**Methods:**

A comprehensive literature search was conducted in PubMed, Embase, Web of Science, and Cochrane Library from inception to August 2025. Study quality was assessed using the NOS scale, AHRQ criteria, and the GRADE framework. Statistical analyses, performed with R 4.5.0 and Stata 14, employed fixed-or random-effects models to calculate pooled odds ratios (ORs) with 95% confidence intervals (CIs). Heterogeneity was explored via sensitivity analyses, and publication bias was evaluated using funnel plots with Begg and Egger tests.

**Results:**

A total of 12 studies involving 2,552 patients were included in this Meta-analysis. The results suggest that the following factors may be associated with AR resistance to treatment with AIT. Gender: male (OR = 1.53, 95% CI: 1.08–2.18); Specific diagnostic antibody aspects: s-IgE/t-IgE ratio (OR = 1.09, 95% CI: 1.02–1.16), sIgE, sIgG4; For cytokines: IL-10, IL-35, TGF-beta, IFN-gamma; On blood parameters: Eosinophil; Immune Cell Aspects:TH2/CD4, TFH2/CD4, CD23 + BNSM, CD23 + BSM, TFR/CD4, TFR/TFH2; Clinical/personal characteristics: demographics, disease severity, allergen type, treatment details, treatment adherence, symptom control, lung function, airway inflammation, inflammatory markers, immunologic markers, imaging markers, environmental and behavioral factors. With respect to the heterogeneity analysis, the heterogeneity of the other analyses was relatively low, except for age and t-IgE levels, where there was significant heterogeneity.

**Conclusion:**

The risk of developing resistance to AIT treatment for AR is closely associated with patient factors including gender, antibodies, cytokines, hematological parameters, clinical/personal characteristics, immune cells, and other indicators.

**Systematic Review Registration:**

PROSPERO CRD420251154551.

## 1 Introduction

Allergic rhinitis (AR) is a chronic inflammatory disease mediated by immunoglobulin E (IgE), characterized by inflammation of the nasal mucosa (1). Clinical manifestations include nasal mucosal congestion, nasal itching and sneezing, etc. (2). While these symptoms are not life-threatening, they can significantly impair a patient's quality of life in the form of sleep disturbances, mood swings, and decreased productivity at work or school (3). Studies have shown that AR is a widespread health problem globally, with prevalence rates on the rise, and is already affecting 5%–50% of the world's population in some way (4). At the same time, it causes productivity losses of between $2 billion and $4 billion per year, resulting in an enormous economic burden (5, 6).

Allergen Immunotherapy (AIT) is largely considered the only therapy that can alleviate AR (7). The mechanisms of AIT treatment are complex and have not yet been fully elucidated. The main mechanism may be a greater shift from a T Helper 2 Cell (Th2) immune response, which is associated with the development of specific diseases, to a T Helper 1 Cell (Th1) immune response, resulting in a better immune homeostasis (8). AIT as a basic therapy for the treatment of respiratory allergic diseases such as AR, providing long-term symptomatic relief and preventing disease progression (9). Currently, subcutaneous immunotherapy (SCIT) and sublingual immunotherapy (SLIT) are the most common treatments for AIT (10). There are advantages and disadvantages to both therapies: SLIT is convenient for home treatment but patient compliance is difficult to monitor; SCIT requires regular visits to the hospital for injections, which facilitates clinical monitoring of compliance but may result in patient absenteeism and loss of income (11). A Meta-analysis study by Jiumei Yang et al. (12) also confirmed that SCIT and SLIT have excellent efficacy and a favorable safety profile in the treatment of AR.

Although the effectiveness of AIT has been confirmed by the results of a large number of real-world studies such as randomized controlled trials (RCTs), it may in some cases result in poor or no response to treatment in patients with AR, i.e., development of resistance, due to the influence of certain risk factors. A review by Maria Angela Tosca et al. (13) concluded that the most common causes of poor SLIT outcomes include diagnostic errors, incorrect allergen dosing and timing, poor quality extracts, comorbidities, impaired immune system function, and inadequate compliance. In contrast, a study by Menno Kiel et al. (14) concluded that low levels of adherence and persistence were the key reasons why SCIT and SLIT for AR did not achieve the desired clinical outcomes and examined the impact of associated risk factors. However, there is still a lack of relatively comprehensive and systematic studies specifically addressing the risk factors for resistance to AIT treatment of allergic rhinitis, and the existing studies hold divergent views and controversial conclusions.

Based on this, the present study used Meta-analysis to systematically evaluate the risk factors for AR resistance in AIT therapy, providing an evidence-based basis for what relevant resistance risk factors need to be prevented when applying this therapy in clinical therapeutic practice.

## 2 Methods

### 2.1 Protocol registration

This study follows the Preferred Reporting Items for Systematic Evaluation and Meta-Analysis (PRISMA) guidelines (45). The protocol for this Meta-analysis is registered with PROSPERO under registration number CRD420251154551.

### 2.2 Literature search

Two researchers (Zhi-qiang Zhang, Jing-yang Li) conducted a comprehensive search of the literature related to risk factors for resistance to AIT treatment of allergic rhinitis in databases such as PubMed, Web of Science, and Cochrane Library. The search strategy is shown in Supplementary Table S1 for the period from the construction of each database to August 2025, using a combination of Medical Subject Headings (MeSH) and free-word searches. English search terms include “Allergic rhinitis, Allergen Immunotherapy, Subcutaneous immunotherapy, Sublingual Immunotherapy”.

### 2.3 Inclusion and exclusion criteria

**Inclusion criteria were:**

Experimental design: cohort studies, class-experimental studies, RCTs, or cross-sectional studies examining risk factors for resistance to AIT for allergic rhinitis.

Study population: patients with AR.

Intervention: SCIT or SLIT treatment.

Main outcome indicators: Age, Gender, Antibody.

Secondary outcome indicator: Cytokines, Hematological parameters, Clinical/personal characteristics, Immune cells and other indicators.

The language of the literature included was English.

**Exclusion criteria included:** (i) reviews, case reports, conference abstracts, commentaries, and letters; (ii) flawed or illogical study design protocols; (iii) incomplete raw data or inability to extract outcome indicators; and (iv) unavailability of full-text literature.

### 2.4 Data collection and quality assessment

Eligible articles were independently screened by two researchers (Zhi-qiang Zhang, Jing-yang Li) based on article titles and abstracts. After further reading of the full text, the literature was again screened to finalize inclusion and extract information, and any differences of opinion were resolved through discussion or consultation with a third party (Xin-hua Zhu).If there is insufficient data, try contacting the newsletter author by e-mail to obtain the missing data.

After the literature was screened and entered, the data were extracted and cross-checked, and the data collected included: (i) general information: first author, date of publication and country, etc.; (ii) detailed information about the trial and control groups (sample size, intervention, gender and age, etc.); and (iii) outcome indicators. All included cohort studies were independently assessed by two researchers (Zhi-qiang Zhang, Jing-yang Li) using the Newcastle-Ottawa Scale (NOS), and the quality of the studies was categorized as high-quality (≥8 points), moderate-quality (5–7 points), or low-quality (<5 points) based on the NOS score. For cross-sectional studies, risk of bias was assessed using the Agency for Healthcare Research Quality (AHRQ) standards. For RCTs, risk of bias was independently assessed using the Cochrane RoB 2 tool, and the quality of literature was assessed using the ROBINS-I scale (Risk of bias in nonrandomized studies of interventions version I) for experimental studies. And data quality was independently assessed and cross-checked using the GRADE data quality assessment tool.

### 2.5 Statistical analysis

Statistical analyses were performed using R 4.5.0 (R Foundation for Statistical Computing, Vienna, Austria), RevMan 5.4 (Cochrane Collaboration, Copenhagen, Denmark), and Stata 14 (Stata Corporation, College Station, TX, USA).

Measurement data were reported as ratio (OR) and 95% confidence interval (CI). The results of each study were tested for heterogeneity by statistical software. Heterogeneity between studies was quantified using the *I*^2^ statistic. If *I*^2^ ≤ 50%, Meta-analysis was performed using a fixed-effects model; conversely, the results of each study were analyzed using a random-effects model, suggesting that there was statistically significant heterogeneity between the results of the studies, and that sensitivity analyses or subgroup analyses would be needed to explore the sources of heterogeneity. Funnel plots and Begg and Egger tests were used to assess publication bias.

## 3 Results

### 3.1 Literature retrieved and characteristics of the included studies

A total of 12,810 articles were retrieved through the preliminary database search, and after removing duplicates, 260 articles remained after two researchers (Zhi-qiang Zhang, Jing-yang Li) read the titles and abstracts for screening; after reading and evaluating the full text, the remaining 12 articles were screened layer by layer and finally included in the analysis (15, 26). The literature was screened as shown in the PRISMA flowchart. A total of 2,552 participants were included in eligible studies, of which 1,324 patients received SCIT, 1,023 patients received SLIT, and 205 patients and healthy individuals did not receive SCIT or SLIT. The basic characteristics of the included studies are shown in Table 1.

**Table 1:** Basic characteristics of the included studies.

Inclusion of literature (first author, date of publication)	Research type	Area	Sample size	Age	Sex (M/F)	Interventions	Highly reactive (test)	Low response (control)	Allergens	Immunotherapy agents
Liu et al. (2020) (15)	Type of experimental research	China	466	7.8 ± 2.5	225/241	SLIT	303	163	Der f	Der f drops
Xie et al. (2021) (16)	Prospective cohort study	China	237	19.9–39.3	126/111	SLIT	45	35	HDM	Der f drops
Lee et al. (2018) (17)	Retrospective cohort study	Korea	304	27.8 ± 11.2	160/144	SCIT	119	185	HDM only or HDM + pollen	Novo-Helisen Depot
Gur Cetinkaya et al. (2020) (18)	Retrospective cohort study	Turkey	261	12.0 ± 3.0	177/84	SCIT	59	202	Grass pollen, etc.	Allergovit 006 Grass
Hoshino et al. (2024) (19)	A randomized controlled study	Japan	140	20–65	44/96	SLIT	43	23	HDM	HDM SLIT tablets
Harintajinda et al. (2025) (20)	Cross-sectional Studies	Thailand	240	21 (11–36)	113/127	SCIT	174	66	HDM	Der p and Der f extracts
Koca Kalkan et al. (2021) (21)	Retrospective cohort study	Turkey	124	Median: 35 (19–77)	52/72	SCIT	48	15	Pollen, etc.	Premixed or single allergen extracts
Wei et al. (2025) (22)	Type of experimental research	China	100	28.5 (18–41)	55/45	SLIT	94	6	HDM	Dust mite drops
Li et al. (2024) (23)	Type of experimental research	China	285	4–14	165/120	SLIT	223	26	HDM	Der f drops
Tu et al. (2023) (24)	Retrospective cohort study	China	98	Median: 21 (9–53)	-	SCIT	69	29	HDM, etc.	Alutard SQ
Wang et al. (2024) (25)	Prospective cohort study	China	72	22–43	29/43	SCIT	49	23	HDM	Semi-depot HDM allergen extracts
Lin et al. (2023) (26)	Type of experimental research	China	225	8.9 ± 2.6	150/75	SCIT	54	16	HDM, etc.	Alutard Der p vaccine

SLIT, sublingual immunotherapy; SCIT, subcutaneous immunotherapy; Der f, dermatophagoides farinae; Der p, dermatophagoides pteronyssinus; HDM, house dust mite.

### 3.2 Quality assessment

Of the 12 included papers, 6 were cohort studies (16–18, 21, 25), 1 was a RCT (19), 1 was a cross-sectional study (20), and 4 were classified as experimental studies (15, 22, 23, 26).

**Figure 1:**
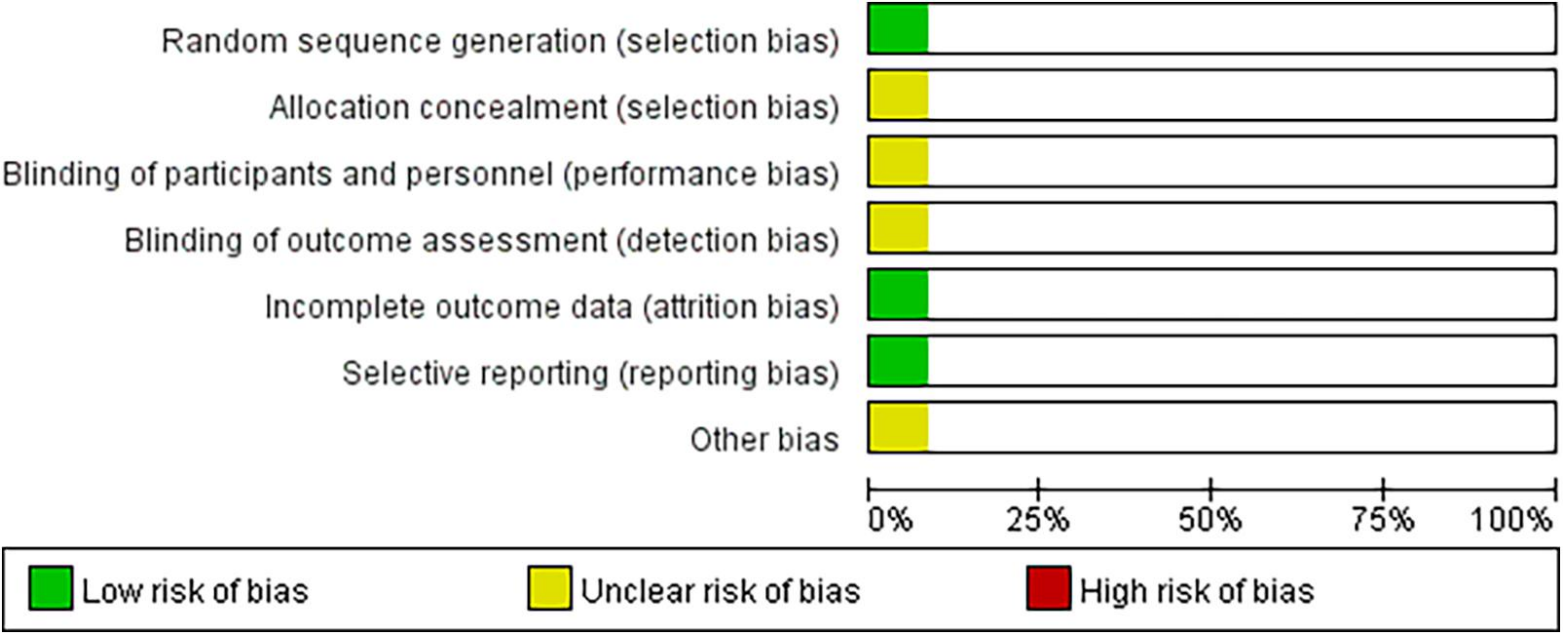
Distribution of risk of bias in the included literature.

**Figure 2:**
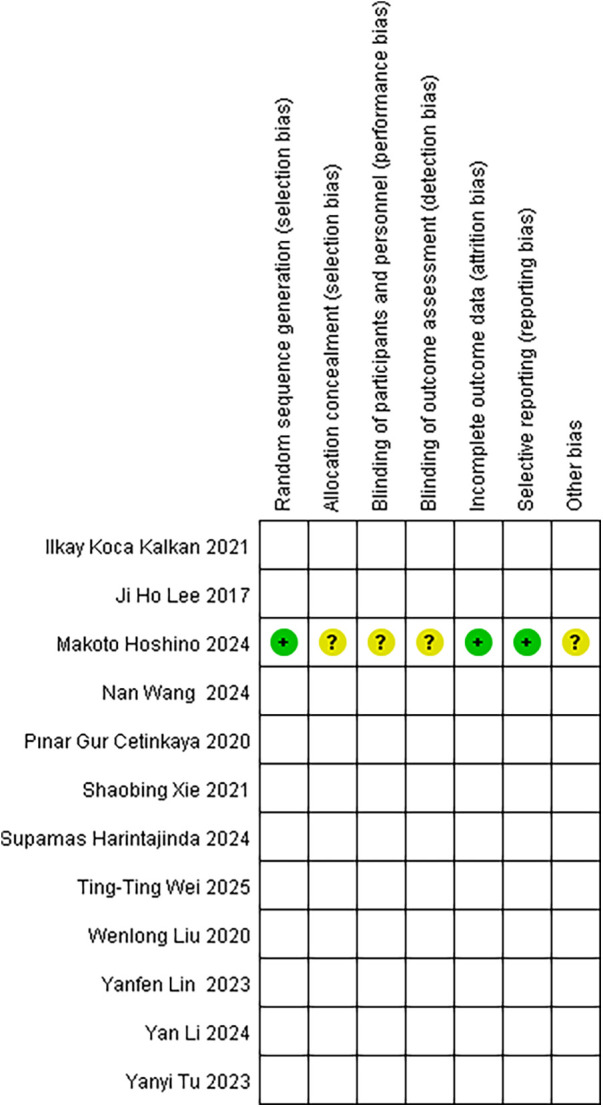
Risk of bias results for the included literature.

All included cohort studies were assessed using the NOS scale. The assessment showed that there were five high-quality studies, only one medium-quality study, and no studies were considered low quality (Table 2). In addition, the cross-sectional studies were evaluated according to AHRQ criteria, and after a detailed review, the results showed a final score between 4 and 7, indicating moderate quality literature and no high-risk studies were included Table 3). The specific risk of bias distribution and outcomes of the RCT are shown in Figures 1, 2, respectively. For the class of experimental studies, one had a serious risk of bias, two had a high risk of bias, and one had a moderate risk of bias (Table 4).

**Table 2:** Quality of included studies based on the Newcastle-Ottawa scale.

Study	Selection (0–4 stars)				Comparability (0–2 stars)	Outcome (0–3 stars)			Total NOS score (0–9)
	Q1	Q2	Q3	Q4	Q5	Q6	Q7	Q8	
Xie et al. (2021) (16)	*	*	*	*	**	*	*	*	9
Lee et al. (2018) (17)	*	*	*	*	*	*	*	*	8
Gur Cetinkaya et al. (2020) (18)	*	*	*	*	**	*	–	*	8
Koca Kalkan et al. (2021) (21)	*	*	*	*	*	*	*	*	8
Tu et al. (2023) (24)	*	-	*	*	*	*	*	*	7
Wang et al. (2024) (25)	*	-	*	*	**	*	*	*	9
Q1: Representativeness of the exposed cohort									
Q2: Selection of the non-exposed cohort									
Q3: Ascertainment of exposure									
Q4: Demonstration that outcome of interest was not present at the start of the study									
Q5: Comparability of cohorts on the basis of the design or analysis									
Q6: Assessment of outcome									
Q7: Was followed up long enough for outcomes to occur									
Q8: Adequacy of follow-up of cohorts									

“*” Represents one point.

**Table 3:** Assessment of the quality of cross-sectional studies based on the criteria recommended by the US agency for healthcare quality and research.

Study	Q1	Q2	Q3	Q4	Q5	Q6	Q7	Q8	Q9	Q10	Q11
Harintajinda et al. (2025) (20)	Yes	Yes	Yes	Unclear	No	Yes	No	No	Ye	Yes	Unclear
Q1: Define the source of information (survey, record review)											
Q2: List the inclusion and exclusion criteria for exposed and unexposed subjects (cases and controls) or refer to previous publications											
Q3: Indicate time period used for identifying patients											
Q4: Indicate whether or not subjects were consecutive if not population-based											
Q5: Indicate if evaluators of subjective components of study were masked to other aspects of the status of the participants											
Q6: Describe any assessments undertaken for quality assurance purposes (e.g., test/retest of primary outcome measurements)											
Q7: Explain any patient exclusion from analysis											
Q8: Describe how confounder was assessed and/or controlled											
Q9: If applicable, explain how missing data were handled in the analysis											
Q10: Summarize patient response rates and completeness of data collection											
Q11: Clarify what follow-up, if any, was expected and the percentage of patients for which incomplete data or follow-up was obtained											
(Yes/No/Unclear)											

**Table 4:** ROBINS-I assessment of study bias for included studies.

Study	Bias Due to Confounding	Bias in Selection of Participants into the Study	Bias in Classification of Interventions	Bias Due to Deviations from Intended Interventions	Bias Due to Missing Data	Bias in Measurement of Outcomes	Bias in Selection of the Reported Results	Overall risk of bias
Liu et al. (2020) (15)	Serious	Low	Low	Moderate	Low	Serious	Low	Serious
Wei et al. (2025) (22)	Critical	Low	Low	Low	Low	Moderate	Low	Critical
Li et al. (2024) (23)	Moderate	Low	Low	Moderate	Moderate	Low	Low	Moderate
Lin et al. (2023) (26)	Moderate	Low	Low	Serious	Serious	Low	Low	Serious

**Figure 3:**
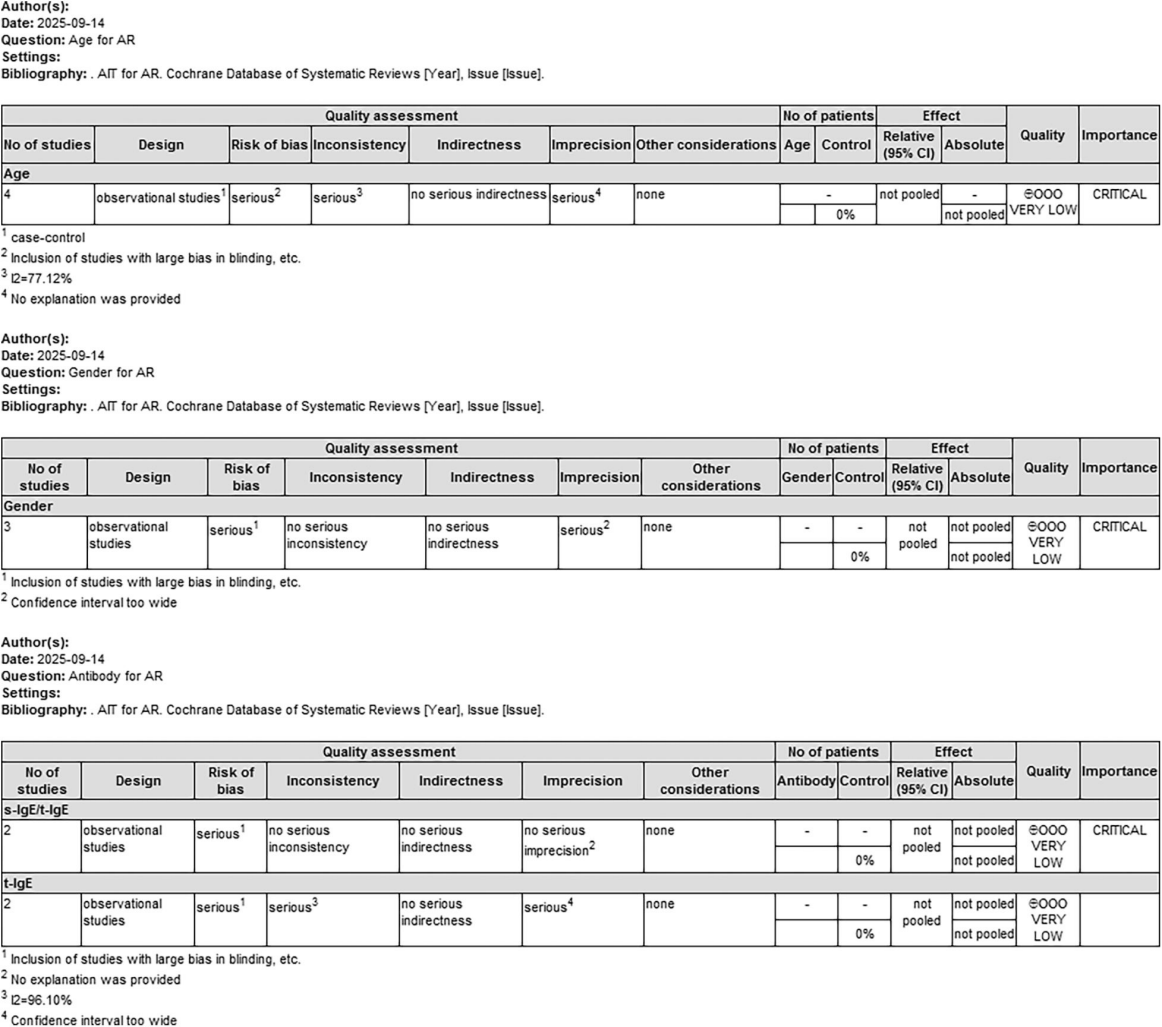
Assessment of the quality of data for inclusion in the literature.

The specific data quality assessment is shown in Figure 3, which shows that the quality of evidence for all four outcomes is very low.

### 3.3 Relationship between age and response to immunotherapy in patients with AR

**Figure 4:**
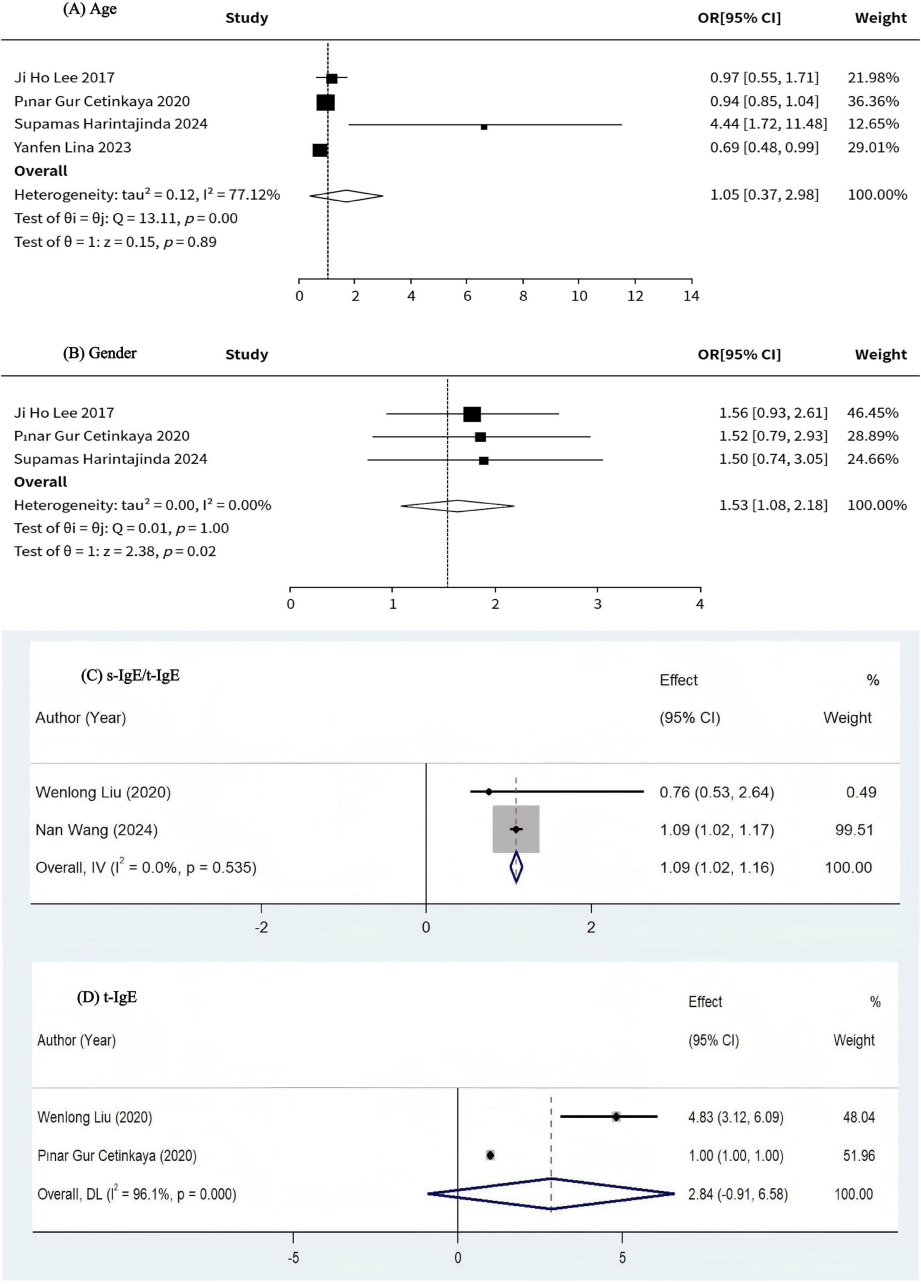
**(A)** Relationship between age and response to immunotherapy in patients with allergic rhinitis. **(B)** Relationship between gender and response to immunotherapy in patients with allergic rhinitis. **(C)** Relationship between s-IgE/t-IgE and response to immunotherapy in patients with allergic rhinitis. **(D)** Relationship between t-IgE and response to immunotherapy in patients with allergic rhinitis.

A total of four studies investigated the relationship between age and response to immunotherapy in patients with AR (17, 18, 20, 26). Meta-analysis results showed no association between age and response to immunotherapy in AR patients (OR = 1.05, 95% CI: 0.37–2.98, *I*^2^ = 77.12%, *P* = 0.89), which was not statistically significant, (Figure 4A as well as Table 5). Given the relatively high heterogeneity of the findings, we used a random effects model for the calculations. Funnel plots revealed asymmetry (Supplementary Figure S1), while the Egger test (*t* = 0.560, *p* = 0.632 > 0.05) showed no evidence of publication bias (Supplementary Table S2). A sensitivity analysis (Supplementary Figure S2) was performed after using the Trim cut-and-patch method on the funnel plots to explore whether the heterogeneity stemmed from the one-by-one exclusion test. The analysis showed that the exclusion of any single study did not significantly affect the results of the study.

**Table 5:** Relationship between age and response to immunotherapy in patients with allergic rhinitis (as OR).

Age	Number of studies	OR	95%CI lower limit	95% CI upper limit	*I* ^2^	*P*-value
ALL	4 (17, 18, 20, 26)	1.05	0.37	2.98	77.12%	0.89
Adults	2 (17, 20)	1.02	0.44	1.59	47.60%	0.167
Children	2 (18, 26)	0.84	0.6	1.08	69.40%	0.071

### 3.4 Relationship between gender and response to immunotherapy in AR patients

A total of four studies investigated the relationship between gender and response to immunotherapy in patients with AR (17, 18, 20, 26). The results of the Meta-analysis showed an association between the gender of AR patients being male and the response to immunotherapy, with male AR patients having a higher clinical response to the application of AIT for the treatment of AR (OR = 1.53, 95% CI:1.08–2.18, *I*^2^ = 0.00%, *P* = 0.02), (Figure 4B as well as Table 6).The heterogeneity test suggested that there was little heterogeneity between the studies, and therefore the fixed-effects model was used for the calculations. The funnel plot was largely symmetrical (Supplementary Figure S3), and the Egger test (*t* = −11.069, *p* = 0.057 > 0.05) showed no evidence of publication bias (Supplementary Table S3).

**Table 6:** Relationship between gender and response to immunotherapy in patients with allergic rhinitis (in terms of OR).

Gender	Number of studies	OR	95%CI lower limit	95% CI upper limit	*I* ^2^	*P*-value
Male	3 (17, 18, 20)	1.53	1.08	2.18	0.00%	0.02
Female	1 (26)	0.297	0.031	2.887	–	–

### 3.5 Relationship between antibodies and immunotherapy response in patients with AR

A total of eight studies investigated the relationship between antibodies and immunotherapy response in AR patients (15–19, 22, 24, 25). Comparing the s-IgE/t-IgE ratio of AR patients in each experiment, the results of Meta-analysis showed that there was an association, positive and statistically significant, between the response to immunotherapy and the s-IgE/t-IgE ratio of AR patients (OR = 1.09, 95% CI: 1.02–1.16, *I*^2^ = 0.00%, *P* = 0.535), (Figure 4C). The heterogeneity test suggested that there was little heterogeneity between the studies, and therefore the fixed-effects model was used for the calculations. The funnel plot (Supplementary Figure S4) showed no evidence of publication bias. Due to the limited number of studies included, meta-analysis results suggest that there may be no correlation between immunotherapy response and serum IgE levels in AR patients (OR = 2.84, 95% CI: −0.91 to 6.58, *I*^2^ = 96.10%, *P* < 0.001), with no statistical significance (Figure 4D). Given the relatively high heterogeneity of the study results, we performed a sensitivity analysis (Supplementary Figure S5) to explore whether the heterogeneity stemmed from the one-by-one exclusion test. The analysis showed that the exclusion of any single study did not significantly affect the results of the study. The funnel plot (Supplementary Figure S6) showed no evidence of publication bias. The combined results of other relevant indicators based on OR effect sizes showed a statistically significant association between s-IgE levels to Der f, Serum HDM-specific IgE, Specific IgE to HDM ≥17.5 kU/L, and response to immunotherapy with a positive correlation. The combined results based on Mean effect sizes showed a statistically significant association between t-IgE, s-IgE of Der f, s-IgE/t-IgE ratios, Serum HDM (house dust mite)-specific IgE, and response to immunotherapy, with a positive correlation. Combined results based on AUC effect sizes showed that s-IgE: 7.4 kU/L, sIgE, sIgG4, pre-treatment sIgG4 level, and pre-treatment sIgG4 level had good discriminatory power in distinguishing immunotherapy responses. The combined results based on r effect sizes showed a statistically significant association between sIgE levels and immunotherapy response, with a positive correlation (Table 7).

**Table 7:** Relationship between antibodies and response to immunotherapy in patients with allergic rhinitis. (a) Relationship between antibodies and response to immunotherapy in patients with allergic rhinitis (in terms of OR).

Antibody	Number of studies	OR	95% CI lower limit	95% CI upper limit	*I* ^2^	*P*-value
s-IgE/t-IgE	2 (15, 25)	1.09	1.02	1.16	0.00%	0.535
t-IgE	2 (15, 18)	2.84	−0.91	6.58	96.10%	<0.001
s-IgE levels to Der f	1 (15)	2.673	1.983	3.249	–	–
sIgE value (kUA/L)	1 (18)	0.995	0.983	1.008	–	–
Serum HDM (house dust mite)–specific IgE (kU/L)	1 (16)	1.893	1.254	2.544	–	–
Specific IgE to HDM ≥17.5 kU/L	1 (17)	1.85	1.01	3.37	–	–

**Table:** (b) Relationship between antibodies and response to immunotherapy in patients with allergic rhinitis (in Mean).

Antibody	Number of studies	Mean	95% CI lower limit	95% CI upper limit	*I* ^2^	*P*-value
t-IgE	1 (15)	321.5	178.5	534	–	–
IgG4	1 (15)	10.4	8.3	11.8	–	–
s-IgE of Der f	1 (15)	27.7	10.7	68.8	–	–
s-IgE/t-IgE ratios	1 (15)	36.1	21.7	42.9	–	–
Serum total IgE (IU/mL)	1 (16)	235.2	102.4	417.5	–	–
Serum HDM (house dust mite)–specific IgE (kU/L)	1 (16)	10.3	4.3	17.5	–	–
s-IgE HDM (kUL)	1 (19)	11.5	3.6	22.2	–	–
t-IgE (IU/mL)	1 (19)	262	77.2	746	–	–

**Table:** (c) Relationship between antibodies and response to immunotherapy in patients with allergic rhinitis (as measured by AUC).

Antibody	Number of studies	AUC	95% CI lower limit	95% CI upper limit	*I* ^2^	*P*-value
Serum HDM (house dust mite)-specific IgE (kU/L)	1 (16)	0.791	0.688	0.894	–	–
s-IgE: 7.4 kU/L	1 (19)	0.84	0.71	0.96	–	–
sIgE	1 (22)	0.839	0.715	0.963	–	–
sIgG4	1 (22)	0.867	0.749	0.985	–	–
pre-treatment sIgG4 level	1 (22)	0.869	0.787	0.928	–	–
sIgG4	1 (22)	0.793	0.677	0.908	–	–
pre-treatment sIgG4 level	1 (22)	0.818	0.728	0.888	–	–

**Table:** (d) Relationship between antibodies and response to immunotherapy in patients with allergic rhinitis (as r).

Antibody	Number of studies	r	95% CI lower limit	95% CI upper limit	*I* ^2^	*P*-value
sIgE (kU/L)	1 (24)	0.29	–	–	–	–
tIgE (kU/L)	1 (24)	−0.01	–	–	–	–
sIgG4 (mgA/L)	1 (24)	−13.44	–	–	–	–
sIgE/tIgE	1 (24)	4.37	–	–	–	–
sIgE/sIgG4	1 (24)	−0.04	–	–	–	–

### 3.6 Relationship between cytokines and immunotherapy response in AR patients

A total of 2 studies investigated the relationship between cytokines and response to immunotherapy in patients with AR (15, 22). Combined results based on OR effect sizes showed a statistically significant positive association between IL-35 levels and response to immunotherapy. The combined results based on Mean effect sizes showed a statistically significant association between levels of IL-10, IL-35, TGF-beta and response to immunotherapy with positive correlation. The combined results based on AUC effect sizes showed that IFN-γ levels had good discriminatory power in distinguishing immunotherapy responses (Table 8).

**Table 8:** Relationship between cytokines and response to immunotherapy in patients with allergic rhinitis. (a) Relationship between cytokines and response to immunotherapy in patients with allergic rhinitis (in terms of OR).

Cytokine	Number of studies	OR	95%CI lower limit	95%CI upper limit	*I* ^2^	*P*-value
IL-10	1 (15)	1.239	0.642	2.745	–	–
IL-35	1 (15)	1.457	1.109	3.265	–	–
TGF-beta	1 (15)	0.875	0.668	2.188	–	–

**Table:** (b) Relationship between cytokines and response to immunotherapy in patients with allergic rhinitis (in Mean).

Cytokine	Number of studies	Mean	95%CI lower limit	95%CI upper limit	*I* ^2^	*P*-value
IL-4 (pg/mL)	1 (15)	3.4	2.1	4.5	–	–
IL-5 (pg/mL)	1 (15)	87.3	45.8	137.8	–	–
IL-13 (pg/mL)	1 (15)	1,256.8	783.1	1,428.3	–	–
IL-12 (pg/mL)	1 (15)	78.2	52.1	124.6	–	–
IFN-*γ* (pg/mL)	1 (15)	245.1	178.3	301.6	–	–
IL-17 (pg/mL)	1 (15)	124.5	86.9	147.3	–	–
IL-10 (pg/mL)	1 (15)	92.5	71.3	101.6	–	–
IL-35 (pg/mL)	1 (15)	187.3	146.5	221.8	–	–
TGF-beta (pg/mL)	1 (15)	192.1	132.5	223.4	–	–

**Table:** (c) Relationship between cytokines and response to immunotherapy in patients with allergic rhinitis (as measured by AUC).

Cytokine	Number of studies	AUC	95%CI lower limit	95%CI upper limit	*I* ^2^	*P*-value
IL-4	1 (22)	0.676	0.479	0.874	–	–
IL-17	1 (22)	0.697	0.495	0.899	–	–
IL-10	1 (22)	0.668	0.465	0.872	–	–
IFN-γ	1 (22)	0.804	0.642	0.966	–	–

### 3.7 Relationship between blood parameters and immunotherapy response in patients with AR

A total of three studies investigated the relationship between blood parameters and response to immunotherapy in patients with AR (15, 16, 18). Combined results based on OR effect sizes showed a positive and statistically significant association between Eosinophil (%) and response to immunotherapy. The combined results based on Mean effect sizes showed no statistically significant association between any of the blood parameters studied and response to immunotherapy. The combined results based on AUC effect sizes showed that none of the blood parameters studied had good discriminatory power in distinguishing immunotherapy responses (Table 9).

**Table 9:** Relationship between hematological parameters and response to immunotherapy in patients with allergic rhinitis. (a) Relationship between hematological parameters and response to immunotherapy in patients with allergic rhinitis (in terms of OR).

Hematologic parameters	Number of studies	OR	95%CI lower limit	95% CI upper limit	*I* ^2^	*P*-value
Blood eosinophil count (10^6/L)	1 (16)	1.219	0.936	1.793	–	–
Blood eosinophil percentage (%)	1 (16)	1.647	0.924	2.098	–	–
Eosinophil (%)	1 (18)	1.146	1.029	1.276	–	–

**Table:** (b) Relationship between hematological parameters and response to immunotherapy in patients with allergic rhinitis (in Mean).

Hematologic parameters	Number of studies	Mean	95%CI lower limit	95% CI upper limit	*I* ^2^	*P*-value
Blood leukocyte count (×10^9^/L)	1 (15)	5.88	5.03	7.53	–	–
Blood neutrophil count (×10^9^/L)	1 (15)	3.07	2.53	3.85	–	–
Blood neutrophil percent (%)	1 (15)	49.1	45.3	57.6	–	–
Blood lymphocyte count (×10^9^/L)	1 (15)	2.07	1.69	2.78	–	–
Blood lymphocyte percent (%)	1 (15)	33.1	29.5	42.8	–	–
Blood eosinophil count (×10^9^/L)	1 (15)	0.21	0.13	0.48	–	–
Blood eosinophil percent (%)	1 (15)	3.65	2.34	8.67	–	–
Blood monocyte count (×10^9^/L)	1 (15)	0.42	0.35	0.56	–	–
Blood monocyte percent (%)	1 (15)	7.64	6.34	9.81	–	–
Blood basophil count (×10^9^/L)	1 (15)	0.06	0.03	0.11	–	–
Blood basophil percent (%)	1 (15)	1.03	0.85	1.04	–	–
Blood eosinophil count (10^6^/L)	1 (15)	313.2	229	397.4	–	–
Blood eosinophil percentage (%)	1 (15)	3.0	1.8	4.2	–	–

**Table:** (c) Relationship between hematological parameters and response to immunotherapy in patients with allergic rhinitis (as AUC).

Hematologic parameters	Number of studies	AUC	95%CI lower limit	95% CI upper limit	*I* ^2^	*P*-value
Blood eosinophil count (10^6/L)	1 (16)	0.687	0.57	0.804	–	–
Blood eosinophil percentage (%)	1 (16)	0.771	0.667	0.875	–	–

### 3.8 Relationship between clinical/personal characteristics of AR patients and response to immunotherapy

A total of 11 studies investigated the relationship between clinical/personal characteristics of AR patients and response to immunotherapy (15–23, 25, 26). The pooled results based on OR effect sizes showed a statistically significant positive correlation between immunotherapy response and the following factors: Parents' educational background, Time of using the air conditioner, Serum MIF level (ng/mL), Duration of immunotherapy ≥3 years, Initiation of SCIT during the grass pollen season, Presence of LR (local reaction) during grass pollen-specific SCIT, No asthma comorbidity, Duration of AIT, and CSMS (combined symptom and medication score). Allergen types: Polysensitized (dust mites and others) were positively associated with immunotherapy response, while severe AR showed a negative association. All correlations were statistically significant. Allergen types: Polysensitized (dust mites and others) showed a positive correlation with immunotherapy response, while severe AR showed a negative correlation, both statistically significant. The combined results based on Mean effect sizes showed that there was no statistically significant association between any of the clinical/personal characteristics of the AR patients studied and immunotherapy response; the combined results based on AUC effect sizes showed that Serum MIF level (ng/mL), WA/Ao, Feno: 19.0 ppb, and EOS were good in differentiating immunotherapy response discriminatory ability in differentiating immunotherapy response. The combined results based on r effect sizes showed that there was an association between Disease duration, Baseline SMS (symptom-medication score) and response to immunotherapy, which was positive and statistically significant (Table 10).

**Table 10:** Relationship between clinical/personal characteristics and response to immunotherapy in patients with allergic rhinitis. (a) Relationship between clinical/personal characteristics and response to immunotherapy in patients with allergic rhinitis (in terms of OR).

Clinical or self-characteristics	Number of studies	OR	95%CI lower limit	95% CI upper limit	*I* ^2^	*P*-value
Parents’ educational background	1 (15)	3.478	1.251	6.095	–	–
Materials for walls	1 (15)	1.569	0.873	2.574	–	–
Time of using the air conditioner	1 (15)	2.156	1.336	3.488	–	–
Serum MIF level (ng/mL)	1 (16)	2.216	1.489	3.197	–	–
Severe AR	1 (17)	0.4	0.23	0.69	–	–
Duration of immunotherapy ≥3 years	1 (17)	7.37	3.5	15.51	–	–
HDM only	1 (17)	1.36	0.77	2.42	–	–
Polysensitization with other pollens	1 (18)	1.252	0.653	2.402	–	–
SPT wheal size (mm)	1 (18)	1.032	0.972	1.095	–	–
Initiation of SCIT in grass pollen season	1 (18)	4.583	1.19	17.658	–	–
Presence of LR(local reaction) during grass pollen–specific SCIT	1 (18)	4.489	2.399	8.397	–	–
Presence of asthma	1 (18)	1.443	0.804	2.592	–	–
Family history of allergy	1 (18)	1.158	0.499	2.689	–	–
No asthma comorbid	1 (20)	2.67	1	7.12	–	–
No other atopic disease (AD, food allergy)	1 (20)	1.53	0.59	3.97	–	–
Polysensitization	1 (20)	1.12	0.55	2.3	–	–
No family history of atopy	1 (20)	1.64	0.82	3.27	–	–
No smoking exposure	1 (20)	0.71	0.34	1.46	–	–
Duration of AR before start SCIT	1 (20)	0.99	0.1	1	–	–
Disease duration	1 (21)	0.892	0.77	1.034	–	–
Occurrence of Local adverse reaction	1 (21)	2.075	0.081	53.376	–	–
Occurrence of Systemic adverse reaction	1 (21)	0.084	0.005	1.507	–	–
Patients using premedication	1 (21)	2.46	0.093	65.032	–	–
Duration of AIT	1 (21)	1.151	1.032	1.283	–	–
CSMS, combined symptom and medication score	1 (25)	1.175	1.023	1.349	–	–
Allergen kinds: Polysensitized (dust mites and others)	1 (26)	15.511	1.319	182.355	–	–
Allergic history: Yes	1 (26)	0.097	0.009	1.095	–	–
Allergic history of family members: Yes	1 (26)	0.523	0.086	3.16	–	–
Passive smoking: Yes	1(26)	0.481	0.087	2.651	–	–
Eczema: Yes	1 (26)	1.635	0.268	9.964	–	–

**Table:** (b) Relationship between clinical/personal characteristics and response to immunotherapy in patients with allergic rhinitis (in Mean).

Clinical or self-characteristics	Number of studies	Mean	95%CI lower limit	95% CI upper limit	*I* ^2^	*P*-value
Age (years)	1 (15)	7.3	6.1	9.2	–	–
BMI	1 (15)	22	18.7	25.3	–	–
ECP (ng/mL)	1 (15)	40.2	5.6	128.4	–	–
Disease duration (year)	1 (16)	4.4	2.7	6.1	–	–
BMI (kg/m2)	1 (16)	22.7	21	24.4	–	–
TNSS (total nasal symptom score)	1 (16)	9	7	11	–	–
VAS (visual analogue scale)	1 (16)	7	5	9	–	–

**Table:** (c) Relationship between clinical/personal characteristics and response to immunotherapy in patients with allergic rhinitis (as measured by AUC).

Clinicalorself-characteristic	Number of studies	AUC	95%CI lower limit	95% CI upper limit	*I* ^2^	*P*-value
Serum MIF level (ng/mL)	1 (16)	0.877	0.803	0.952	–	–
prebronchodilator FEV1%	1 (19)	0.76	0.59	0.92	–	–
WA/Ao	1 (19)	0.83	0.69	0.97	–	–
Feno: 19.0 ppb	1 (19)	0.89	0.81	0.98	–	–
EOS	1 (22)	0.802	0.62	0.985	–	–

**Table:** (d) Relationship between clinical/personal characteristics and response to immunotherapy in patients with allergic rhinitis (indexed by r).

Clinicalorself-characteristic	Number of studies	r	95%CI lower limit	95% CI upper limit	*I* ^2^	*P*-value
Disease duration	1 (23)	0.35	–	–	–	–
Baseline SMS(symptom-medication score)	1 (23)	0.67	–	–	–	–

### 3.9 Relationship between immune cells and immunotherapy response in AR patients

A total of 1 study investigated the relationship between immune cells and immunotherapy response in AR patients (25). Combined results based on OR effect sizes showed a statistically significant association between T_H_2/CD4, T_FH_2/CD4, CD23 ^+^ B_NSM,_ CD23 ^+^ B_SM_ and response to immunotherapy with a positive correlation and a negative correlation for T_FR_/CD4, T_FR_/T_FH_2 (Table 11).

**Table 11:** Relationship between immune cells and response to immunotherapy in patients with allergic rhinitis (in terms of OR).

Immune cell	Number of studies	OR	95%CI lower limit	95% CI upper limit	*I* ^2^	*P*-value
T_H_2/CD4	1 (25)	1.164	1.013	1.337	–	–
T_FH_2/CD4	1 (25)	1.134	1.026	1.253	–	–
T_FR_/CD4	1 (25)	0.801	0.707	0.908	–	–
T_FR_/T_FH_2	1 (25)	0.849	0.776	0.929	–	–
CD23 ^+^ B_NSM_	1 (25)	1.207	1.054	1.383	–	–
CD23 ^+^ B_SM_	1 (25)	1.214	1.074	1.372	–	–

### 3.10 Relationship between some specific clinical and laboratory markers and response to immunotherapy in AR patients

A total of 2 studies investigated the relationship between a number of specific clinical and laboratory markers and response to immunotherapy in patients with AR (18, 19). The combined results based on Median effect sizes showed that there was no statistically significant association between Age at the first dose of grass pollen SCIT, SPT wheal size, sIgE value, Total IgE and response to immunotherapy. The combined results based on beta effect sizes showed a positive and statistically significant association between Prebronchodilator FEV1, Feno, s-IgE HDM and response to immunotherapy Table 12).

**Table 12:** Relationship between other indicators and response to immunotherapy in patients with allergic rhinitis. (a) Relationship between other indicators and response to immunotherapy in patients with allergic rhinitis (in Median).

Indicators	Number of studies	Median	95%CI lower limit	95% CI upper limit	I^2^	*P*-value
Age at the first dose of grass pollen SCIT (years)	1 (18)	12.3	8.8	14.3	–	–
SPT wheal size (mm)	1 (18)	13.5	10	17.7	–	–
sIgE value (kUA/L)	1 (18)	44.7	24.7	98.7	–	–
Total IgE (kU/L)	1 (18)	254.5	110.7	558	–	–

**Table:** (b) Relationship between other indicators and response to immunotherapy in patients with allergic rhinitis (in terms of *β*).

Indicators	Number of studies	*β*	95%CI lower limit	95% CI upper limit	*I* ^2^	*P*-value
Prebronchodilator FEV1 (% predicted)	1 (19)	0.043	0.006	0.079	–	–
WA/Ao (%)	1 (19)	0.001	−0.006	0.011	–	–
Feno (ppb)	1 (19)	0.015	0.009	0.021	–	–
s-IgE HDM (kU/L)	1 (19)	0.018	0.002	0.034	–	–

## 4 Discussion

### 4.1 Main findings

A total of 12 studies involving 2,552 participants were included in this Meta-analysis. To the best of our knowledge, this is the first comprehensive exploration of risk factors for the risk of AR resistance to AIT therapy, and a systematic analysis identified multiple risk factors that may increase the risk of AR resistance to AIT therapy. These risk factors include gender, antibodies, cytokines, hematological parameters, clinical/personal characteristics, immune cells, and other indicators. These findings have important clinical implications and may provide guidance for early prevention of resistance to AIT therapy for AR as well as evidence in evidence-based medicine.

### 4.2 Interpretation of findings

In terms of the relationship between gender and resistance to AIT for the treatment of AR, our findings indicated that male patients were more at risk of resistance to AIT for the treatment of AR than females. In addition, the results of this study had little heterogeneity and after Egger's test with sensitivity analysis, the results indicated that this conclusion had high reliability. A study conducted by Ji Ho Lee et al (27), on the other hand, showed that male patients had a greater chance of nonadherence when receiving AIT for AR, and nonadherence leads to insufficient regimens, so that AIT has a worse remission effect on male AR patients than on females, which is contrary to the findings of our study. In addition, a previous study by Marco De Carli et al. (28) noted that no data have been generated to date on the differences in response to AIT between male and female AR patients and that further studies are needed to prove this hypothesis, and our findings provide some evidence in this regard.

In terms of the relationship between antibodies and resistance to AIT for AR, our findings indicated that decreased s-IgE/t-IgE, sIgE, sIgG4 in AR patients increased the risk of developing resistance to AIT for AR.s-IgE binds to high-affinity receptors (Fc*ε*RI) on the surface of mast cells and basophils via its Fc segment, sensitizing the organism (29). A transient rise in s-IgE in AR patients during AIT treatment indicates activation of the immune system, which begins to function, and serological allergen sIgE testing has long been recognized as an effective diagnostic method for allergic diseases as well (30). Therefore, s-IgE is a very important indicator for monitoring the efficacy of AIT in the treatment of AR.Previous findings of Gulbin Bingol Karakoc et al. (31) showed a significant positive correlation between s-IgE/t-IgE ratio and rhinitis symptom scores (RSS), visual analogue scales (VAS) in patients with AR, which is the same conclusion of our study. This shows that s-IgE/t-IgE can be a potential predictor and evaluator for AR patients treated with AIT.IgG4 activates sensitization by removing free allergens and blocking IgE antibodies so that IgE cannot trigger Fc receptors. In addition, IgG4 prevents mast cell activation via FcγIII and inhibits degranulation and inflammatory mediator release, thereby alleviating allergic symptoms in AR patients (32). Our results showed no association between t-IgE and immunotherapy response, suggesting that t-IgE does not accurately reflect the body's allergic status, which may be due to the fact that they are often affected by a variety of nonspecific factors, such as race, age, gender, environment, and parasitic infections (33).

Regarding the relationship between cytokines and resistance to AIT for AR, our findings indicated that decreased IL-10, IL-35, TGF-beta, and IFN-gamma in AR patients increased the risk of developing resistance to AIT for AR. IL-10 inhibits IgE production by phosphorylating IL-10R on Treg cells via Janus kinase 1 (JAK1), which promotes the proliferation and differentiation of Treg cells (34). IL-10 also inhibits IgE by enhancing IgG4-producing switch-like B cells (35). Mohamed H. Shamjd et al. (36) demonstrated the ability of IL-35 to inhibit the ILC2-mediated pro-allergic type II immune response and to promote immune tolerance and alleviate symptoms associated with AR patients by inducing iT35 cells. The role of TGF-beta in the treatment of AR with AIT is similar to that of IL-10 and IL-35, both of which are secreted by Treg cells and play a role in suppressing the Th2 immune response and inducing immune tolerance (36). IFN-γ inhibits the synthesis of Th2 interleukins, including IL-4, IL-5, and IL-13, thereby affecting key aspects of the sensitization process and relieving patients' AR symptoms (37). Therefore, a decrease in the secretion of these cytokines mentioned above may weaken AIT-induced immune tolerance, leading to a decrease in therapeutic efficacy or the emergence of drug resistance.

In terms of the relationship between blood parameters and resistance to AIT therapy for AR, our findings indicated that a decrease in Eosinophil in AR patients increases the risk of developing resistance to AIT therapy for AR. In contrast, most previous studies have concluded that a decrease in Eosinophil is instead an indication of good efficacy of AIT in treating patients with AR (38, 39). The results of the studies we included in the literature are contradictory, we speculate that this may be related to the following reasons: (1) Differences in baseline patient characteristics across included studies (e.g., age, allergen type, concomitant asthma status); (2) Heterogeneity in the timing and methods of eosinophil testing; (3) Differences in immunomodulatory pathways due to variations in AIT type (SCIT/SLIT) and treatment duration; (4) Potential inconsistencies in eosinophil responses between local tissues and peripheral blood. Further research is needed to explore and confirm the relationships among these factors.

Regarding the relationship between clinical/personal characteristics and AIT treatment resistance in AR, our findings indicate that: AR patients' parents' educational background, duration of air conditioner use, serum MIF level (ng/mL), immunotherapy duration ≥3 years, initiation of SCIT during grass pollen season, presence of LR (local reaction) during grass pollen-specific SCIT, absence of asthma comorbidity, duration of AIT, CSMS (combined symptom and medication score), allergen types: Polysensitized (dust mites and others), WA/Ao, Feno: 19.0 ppb, Eosinophil count, Disease duration, and Baseline SMS (symptom-medication score) are associated with an increased risk of developing resistance to AIT treatment for AR. Conversely, elevated levels of Severe AR are associated with an increased risk. Serum MIF levels are elevated in poor responders, and serum MIF manipulates macrophages, promotes the Th2 immune response, and triggers allergic reactions including IgE production and histamine release (16). Martin Penagos et al. (40) analyzed 3 RCTs of AIT for AR and concluded that Duration of immunotherapy ≥3 years has a long term efficacy in AR and that this efficacy continues for at least 2–3 years after discontinuation of AIT. The findings of this study are the same as ours and strongly suggest that Duration of immunotherapy ≤3 years is a risk factor for AR treated with AIT, the reason for which we speculate may be related to the fact that prolonged immunotherapy can build a durable immune tolerance. For CSMS, Phichayut Phinyod et al. (41) included six studies in a Meta-analysis, and their results showed a trend of decreasing CSMS in both groups after treatment, which is contrary to the results of our included studies, but their results showed no statistical significance.

Regarding the relationship between immune cells and resistance to AIT for AR, our findings indicated that decreased T_H_2/CD4, T_FH_2/CD4, CD23 ^+^ B_NSM_, CD23 ^+^ B_SM_ in AR patients increased the risk of developing resistance to AIT for AR, whereas increased T_FR_/CD4, T_FR_/T_FH_2 increased this risk. Nan Wang et al. (25) conducted a detailed study of T and B cell subsets from AR patients who had used AIT therapy and found that in AR patients T_FH_2 promotes IL-4-mediated IgE production, that CD23 + B_NSM_ acts as a low affinity receptor for IgE and promotes IgE synthesis, and that T_FR_ cells inhibit this process by secreting IL-10.

In terms of the relationship between some specific clinical and laboratory indicators and resistance to AIT for AR treatment, our findings indicated that a decrease in Prebronchodilator FEV1, Feno, s-IgE HDM in AR patients increases the risk of developing resistance to AIT for AR Prebronchodilator. Reduction in FEV1 may be associated with worsening of AR, and a study by Juan Liu et al. (42) also showed improvement in FEV1% in patients after receiving immunotherapy. Therefore, a reduction in the patient's FEV1% is likely to suggest a risk of resistance to AIT treatment of AR. In addition, it has been demonstrated that Feno can reflect the level of airway inflammation in asthma patients with high levels of NO in their peripheral blood, which can be used as an inflammatory marker for asthma (43). According to the theory of “unified airway”, the respiratory tract is regarded as a morphological and functional whole (44). Therefore, it is highly likely that Feno is also a risk factor reflecting poor outcomes in AR patients, but there are fewer relevant studies and further validation is needed.

### 4.3 Strengths of this study

First, this study is the first systematic evaluation and Meta-analysis to comprehensively assess the risk factors for AR resistance in AIT therapy, synthesizing previous relevant studies and incorporating many high-quality studies to compensate for the lack of comprehensive evidence in this line of research. Secondly, this study covers multifaceted types of risk factors, including individual patient characteristics, clinical factors, hematological parameters and immunological factors, providing multifaceted evidence-based support for clinical practice. In addition, the important findings of this study were rigorously validated for bias and robustness. For results with asymmetry, we synthesized the quantitative assessment of publication bias using Egger's linear regression test and further applied the Trim cut-and-fill method to estimate the impact of potential missing studies. And all the sensitivity analyses were conducted on the important factors, and the results showed that the main findings remained stable in the sensitivity analyses, further supporting the reliability of the findings.

### 4.4 Limitations of this study

However, despite these advantages, our Meta-analysis inevitably suffers from several limitations. First, there was a high degree of heterogeneity among the included studies for some outcomes, and after sensitivity analyses, it was found that some of the heterogeneity may have stemmed from inconsistencies in AIT regimens (SCIT or SLIT) or allergen types. Despite extensive sensitivity analyses, we encountered some unexplained heterogeneity. There may be other sources of heterogeneity in the results of our study, such as inconsistencies in the criteria used to diagnose AR, medications used, and dosages administered, which will require future studies to further assess the sources of heterogeneity and standardize the AIT protocols to reduce inter-study heterogeneity by further maintaining consistency in vaccine type, dosage administered, frequency, and duration. Second, there are limitations in the data sources included in the study, and our findings are based primarily on Asian population data. Due to differences between geographic regions in potential confounders such as environmental exposure, genetic background, and lifestyle, these factors may influence the efficacy performance of AIT in allergic rhinitis. Therefore, the findings of the current study should be cautiously generalized to populations outside of Asia. Future multinational studies covering a wider range of regions and populations are needed to further validate the association between the above factors and AIT resistance to enhance the validity and generalizability of the findings. Due to the limited number of included studies, we were unable to fully explore certain potential sources of heterogeneity (e.g., differences in AIT protocols or allergen types) through subgroup analyses or meta-regression. Another significant limitation of this analysis is that for certain exploratory factors—particularly specific antibody markers—we were unable to conduct a thorough quantitative assessment of heterogeneity and perform publication bias tests due to the limited number of original studies available for inclusion. Consequently, findings regarding the association between these factors and AIT efficacy should be regarded as preliminary, hypothesis-generating conclusions whose robustness requires validation through future high-quality studies. As this study was designed as a retrospective Meta-analysis and the data used were mainly derived from published cross-sectional studies or cohort studies, etc., and the inclusion of literature for many of the outcome metrics was scarce, a causal relationship between some of the stated risk factors and AR resistance to AIT treatment could not be definitively inferred, but only a correlation could be hypothesized.

## 5 Conclusion

The results of this latest Meta-analysis suggest that the risk of developing resistance to AIT for AR is strongly associated with the patient's gender, antibodies, cytokines, blood parameters, clinical/personal characteristics, immune cells, and a number of other indicators. However, we also observed significant heterogeneity in our experiments, but the reason for this is unknown, and further studies are needed to explore the underlying mechanisms leading to this phenomenon as a way to further validate the results of this Meta-analysis.

## Data Availability

The original contributions presented in the study are included in the article/Supplementary Material, further inquiries can be directed to the corresponding author.

## Supplementary material

Supplementary Figure S1 Funnel plot of the relationship between age and response to immunotherapy in patients with allergic rhinitis.

Supplementary Figure S2 Sensitivity analysis of the relationship between age and response to immunotherapy in patients with allergic rhinitis.

Supplementary Figure S3 Funnel plot of the relationship between gender and response to immunotherapy in patients with allergic rhinitis.

Supplementary Figure S4 Funnel plot of the relationship between s-IgE/t-IgE and response to immunotherapy in patients with allergic rhinitis.

Supplementary Figure S5 Sensitivity analysis of the relationship between t-IgE and response to immunotherapy in patients with allergic rhinitis.

Supplementary Figure S6 Funnel plot of the relationship between t-IgE and response to immunotherapy in patients with allergic rhinitis.
