# Supplementary material for: Risk factors for drug resistance in allergen immunotherapy for allergic rhinitis: a systematic review and meta-analysis
Source: Front Allergy. 2026 Jan 23;6:1743260. doi: 10.3389/falgy.2025.1743260 (PMC12876254; doi:10.3389/falgy.2025.1743260)
Supplement: Supplementary Figure S1 — Funnel plot of the relationship between age and response to immunotherapy in patients with allergic rhinitis. [file Datasheet1.pdf]

CARE
case report guidelines

CARE Checklist of information to include when writing a case report

(cc) BY-NC-ND

✓

Topic	Item	Checklist item description	Reported on Line
Title	1	The diagnosis or intervention of primary focus followed by the words "case report"	p1, Line 2
Key Words	2	2 to 5 key words that identify diagnoses or interventions in this case report, including "case report"	p4. Line 2.
Abstract (no references)	3a	Introduction: What is unique about this case and what does it add to the scientific literature?	p3, Line 2-4
	3b	Main symptoms and/or important clinical findings	p3, Line 9-16
	3c	The main diagnoses, therapeutic interventions, and outcomes	p3, Line 9-16
	3d	Conclusion—What is the main "take-away" lesson(s) from this case?	p3, Line 17-18
Introduction	4	One or two paragraphs summarizing why this case is unique (**may include references**)	p7. Line 6-7
Patient Information	5a	De-identified patient specific information.	p9, Line 16-18
	5b	Primary concerns and symptoms of the patient.	
	5c	Medical, family, and psycho-social history including relevant genetic information	
	5d	Relevant past interventions with outcomes	
Clinical Findings	6	Describe significant physical examination (PE) and important clinical findings.	
Timeline	7	Historical and current information from this episode of care organized as a timeline	
Diagnostic Assessment	8a	Diagnostic testing (such as PE, laboratory testing, imaging, surveys).	
	8b	Diagnostic challenges (such as access to testing, financial, or cultural)	
	8c	Diagnosis (including other diagnoses considered)	
	8d	Prognosis (such as staging in oncology) where applicable	
Therapeutic Intervention	9a	Types of therapeutic intervention (such as pharmacologic, surgical, preventive, self-care)	p8. Line 18 - p9. Line 3
	9b	Administration of therapeutic intervention (such as dosage, strength, duration)	p8. Line 18 - p9. Line 3
	9c	Changes in therapeutic intervention (with rationale)	
Follow-up and Outcomes	10a	Clinician and patient-assessed outcomes (if available)	p11 - p12
	10b	Important follow-up diagnostic and other test results	
	10c	Intervention adherence and tolerability (How was this assessed?)	
	10d	Adverse and unanticipated events	
Discussion	11a	A scientific discussion of the strengths AND limitations associated with this case report	p17, Line 12 - p18. Line 2
	11b	Discussion of the relevant medical literature **with references**.	p15. Line 4 - p16. Line 3
	11c	The scientific rationale for any conclusions (including assessment of possible causes)	p15, Line 3 - p17. Line 11
	11d	The primary "take-away" lessons of this case report (without references) in a one paragraph conclusion	p18, Line 3-8
Patient Perspective	12	The patient should share their perspective in one to two paragraphs on the treatment(s) they received	
Informed Consent	13	Did the patient give informed consent? Please provide if requested	Yes ☐ No ☑
