# Supplementary figures and images for: Risk factors for drug resistance in allergen immunotherapy for allergic rhinitis: a systematic review and meta-analysis
Source: Front Allergy. 2026 Jan 23;6:1743260. doi: 10.3389/falgy.2025.1743260 (PMC12876254; doi:10.3389/falgy.2025.1743260)

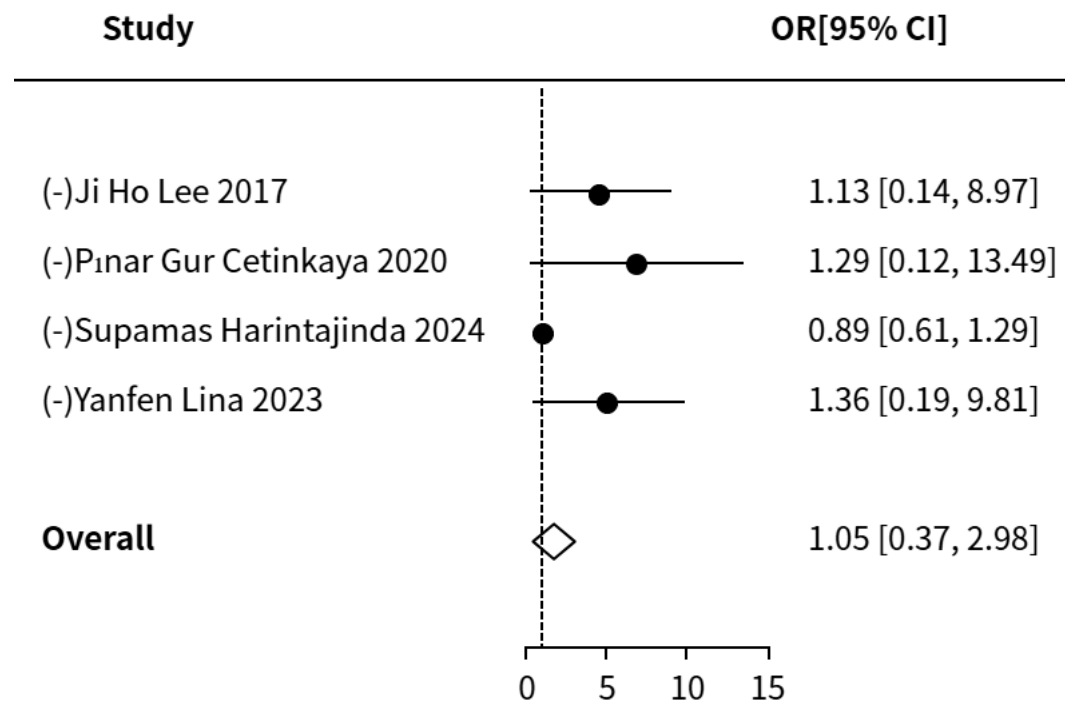

Funnel plot with pseudo 95% confidence limits

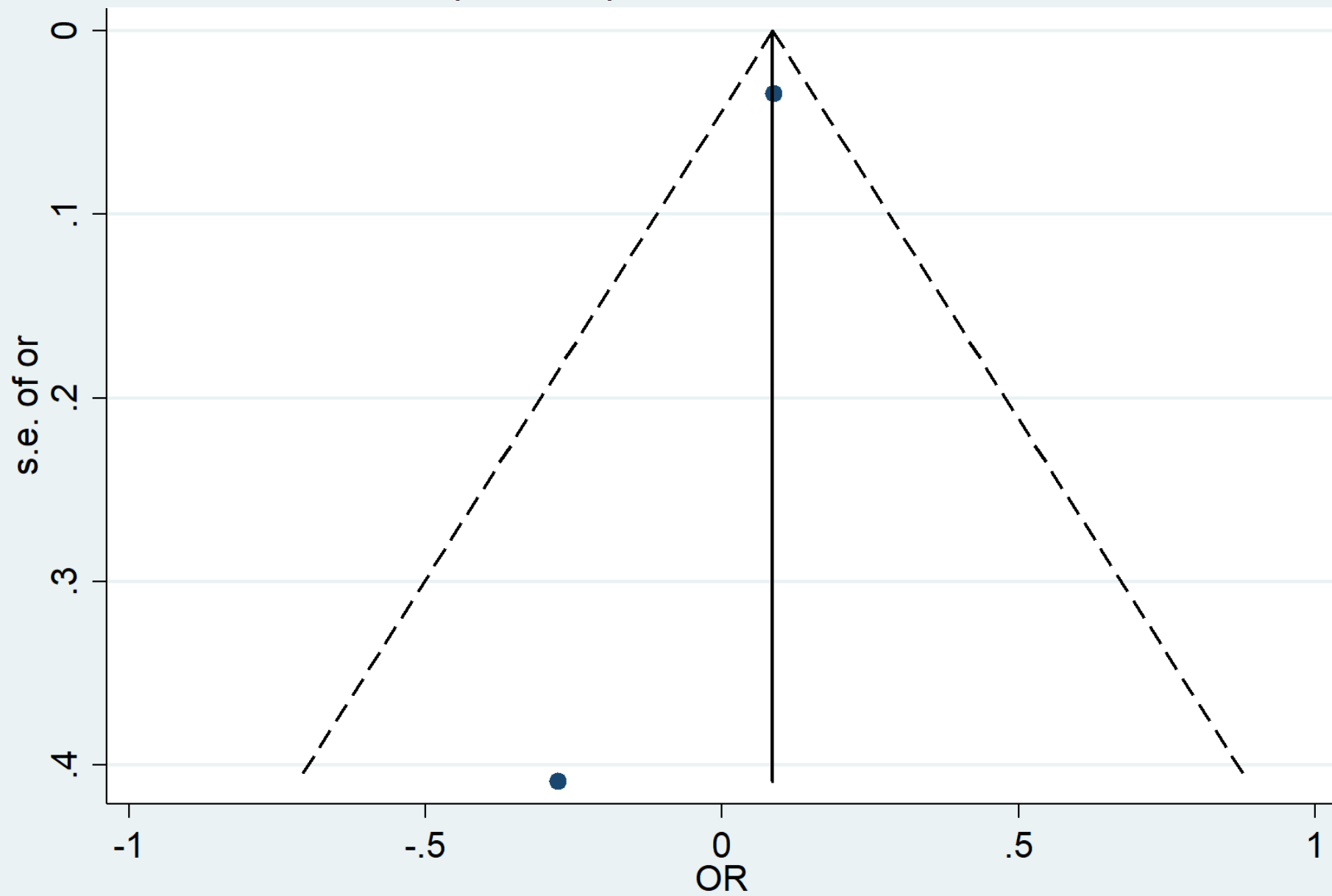

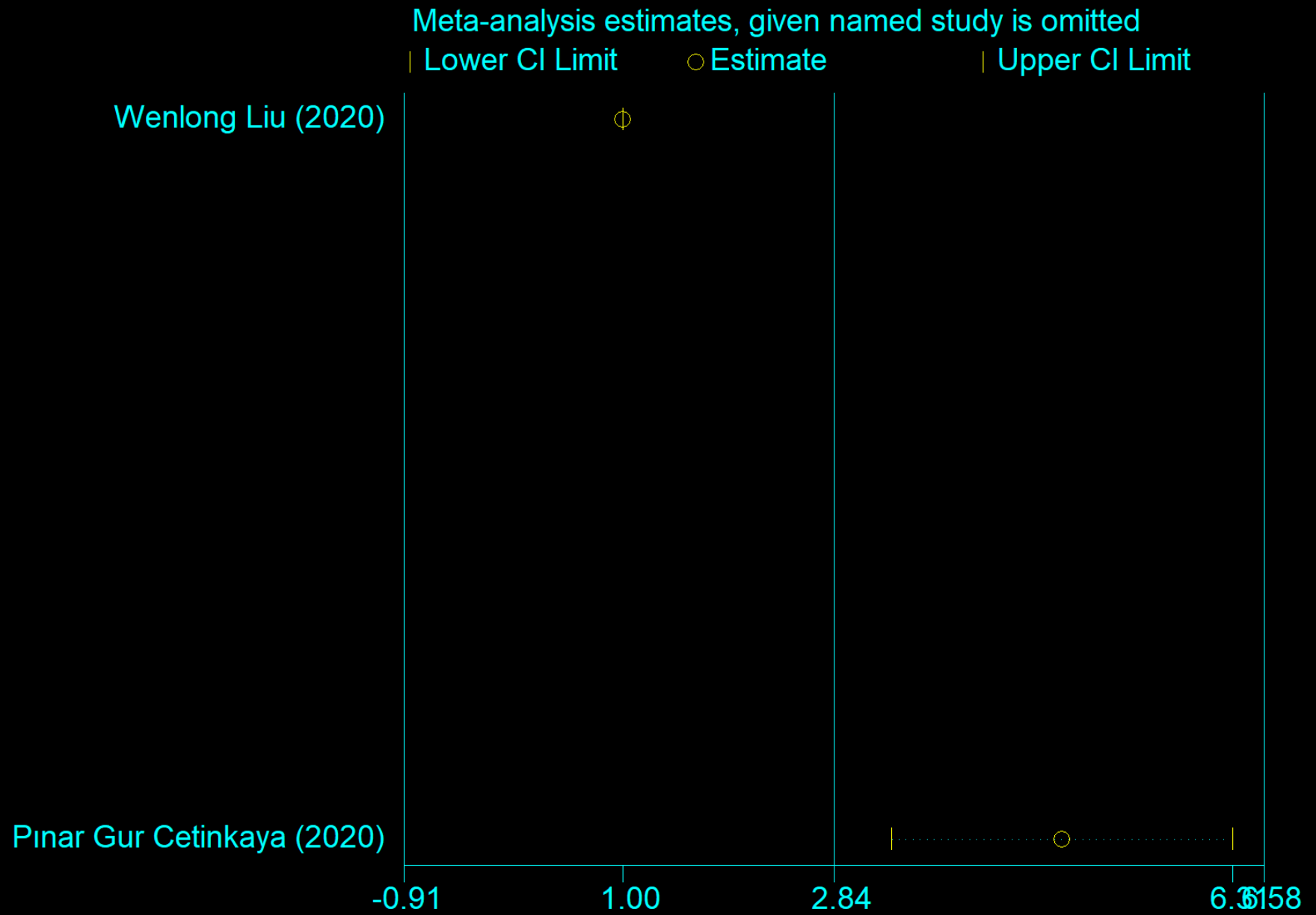

Funnel plot with pseudo 95% confidence limits

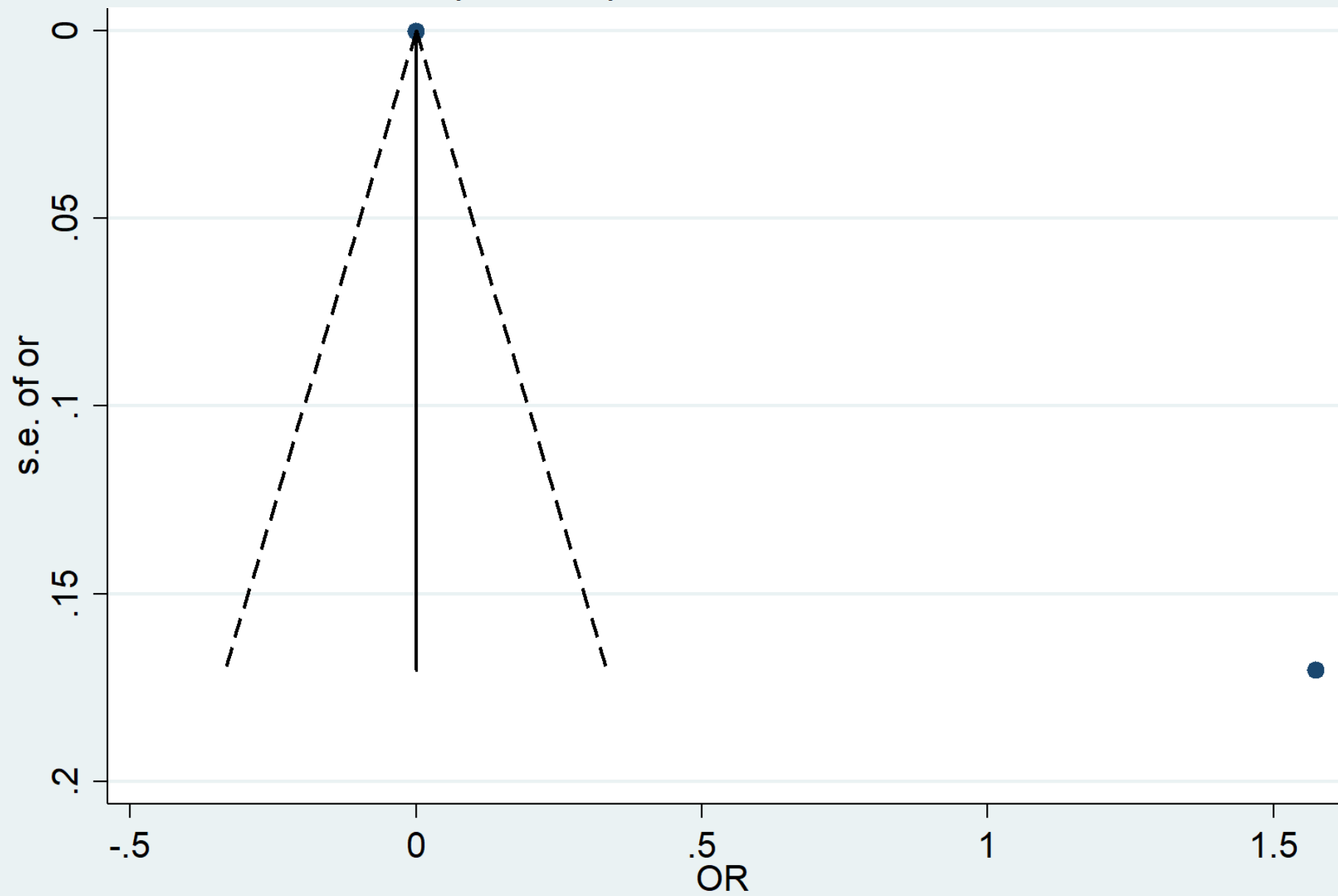

Supplement: Supplementary Figure S2 — Sensitivity analysis of the relationship between age and response to immunotherapy in patients with allergic rhinitis. [file Datasheet2.pdf]

Funnel Chart

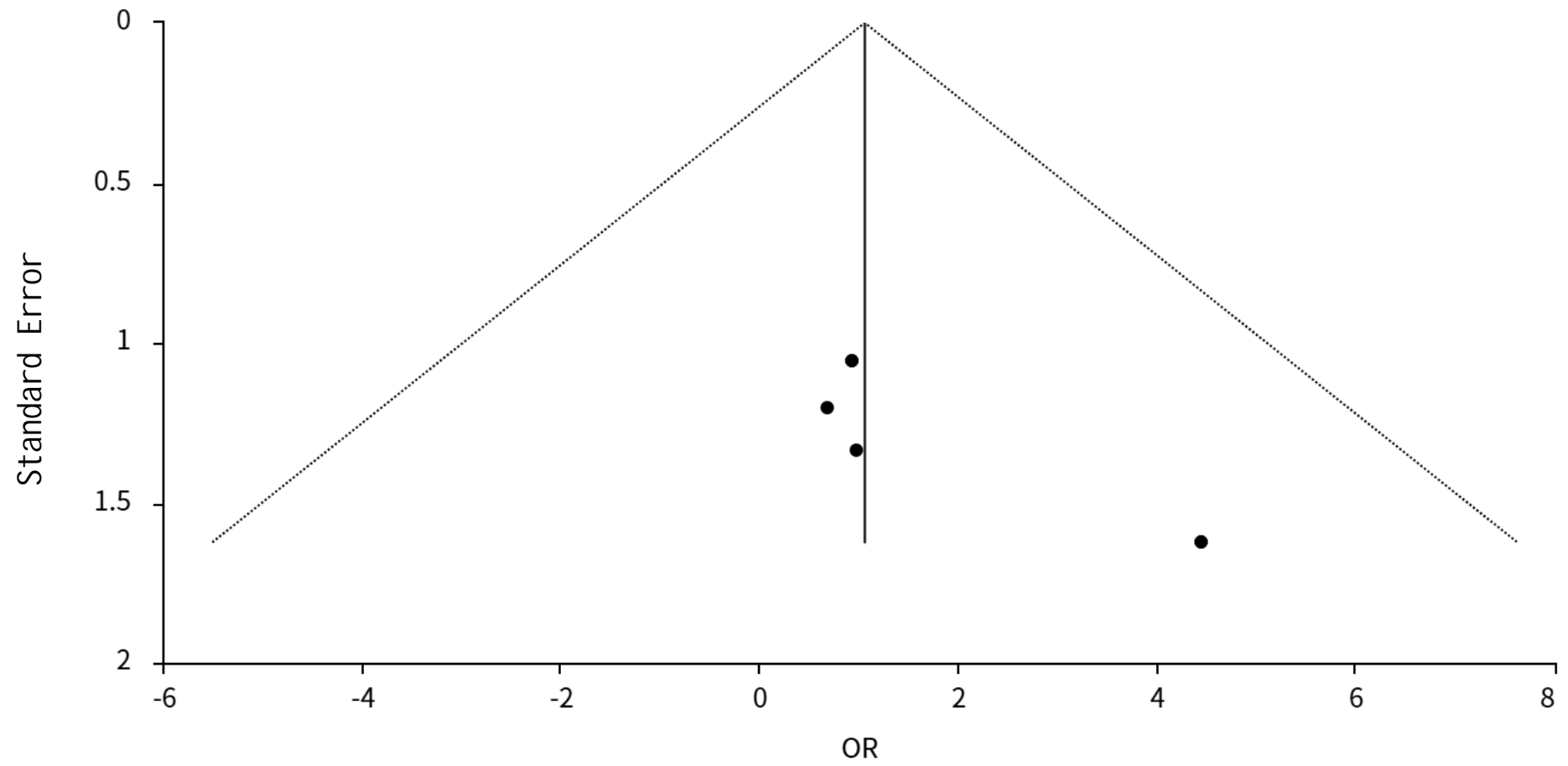

漏斗图

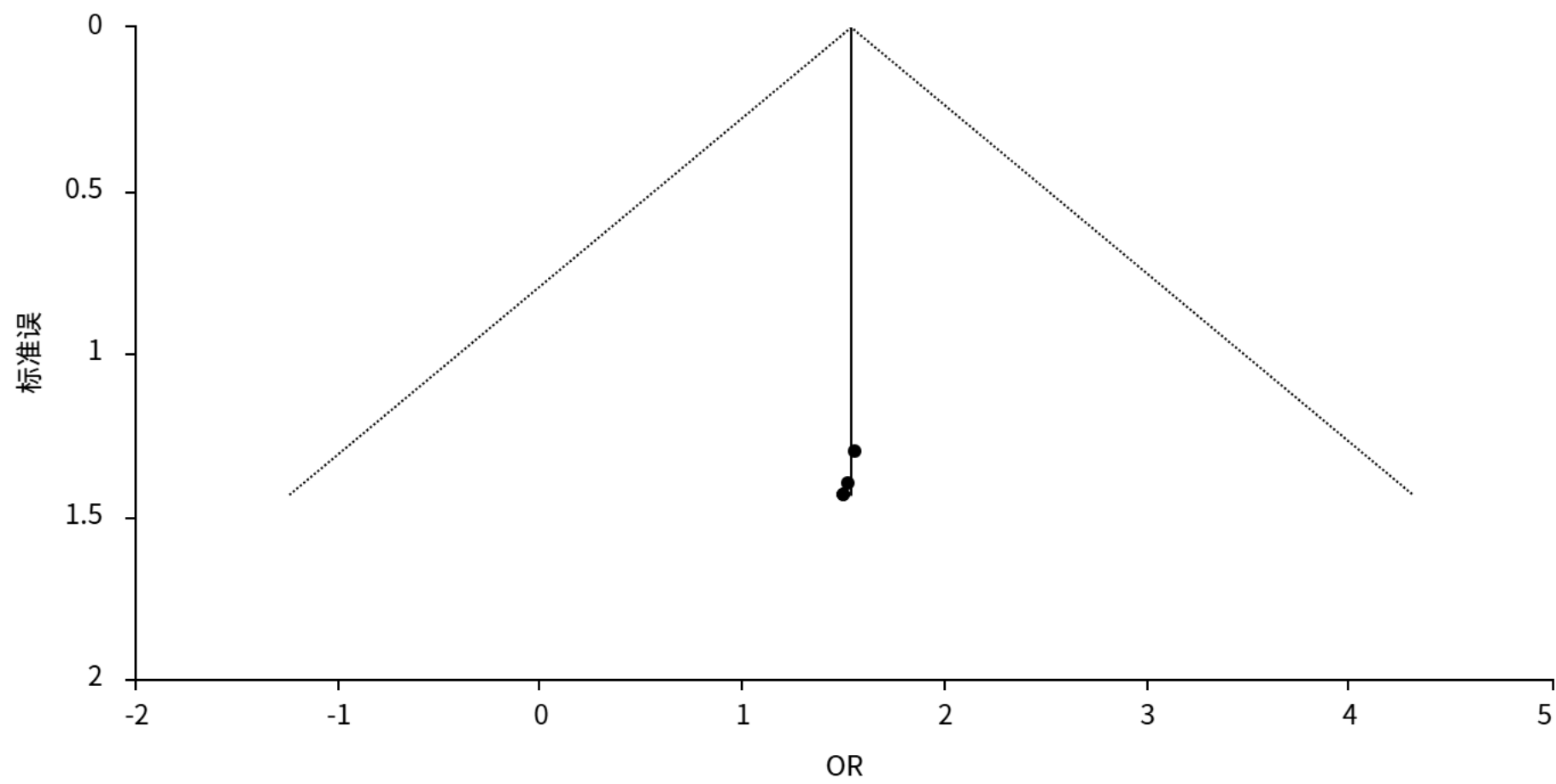

Supplement: Supplementary Figure S3 — Funnel plot of the relationship between gender and response to immunotherapy in patients with allergic rhinitis. [file Datasheet3.pdf]

Funnel plot with pseudo 95% confidence limits

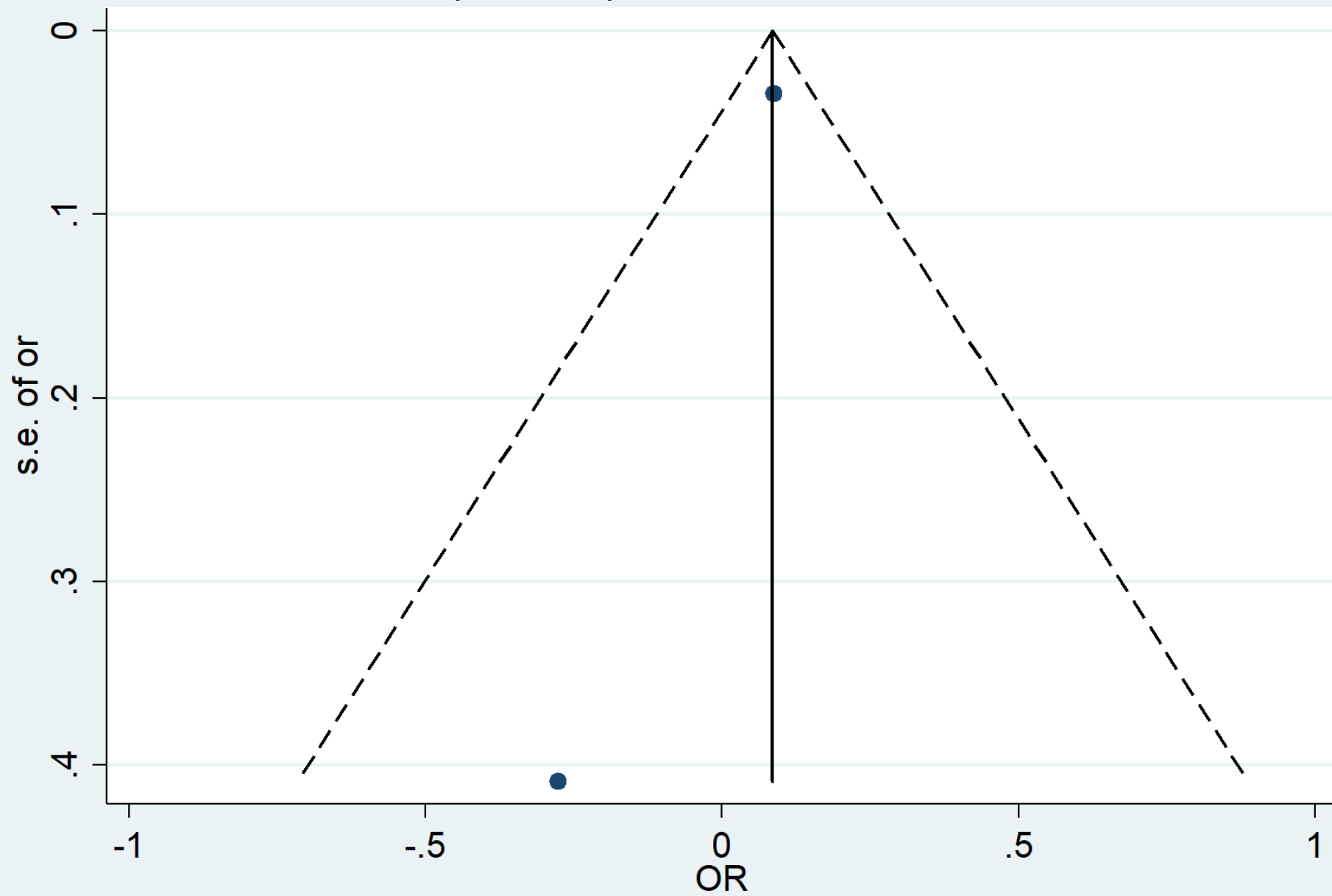

Supplement: Supplementary Figure S4 — Funnel plot of the relationship between s-IgE/t-IgE and response to immunotherapy in patients with allergic rhinitis. [file Datasheet4.pdf]

Funnel plot with pseudo 95% confidence limits

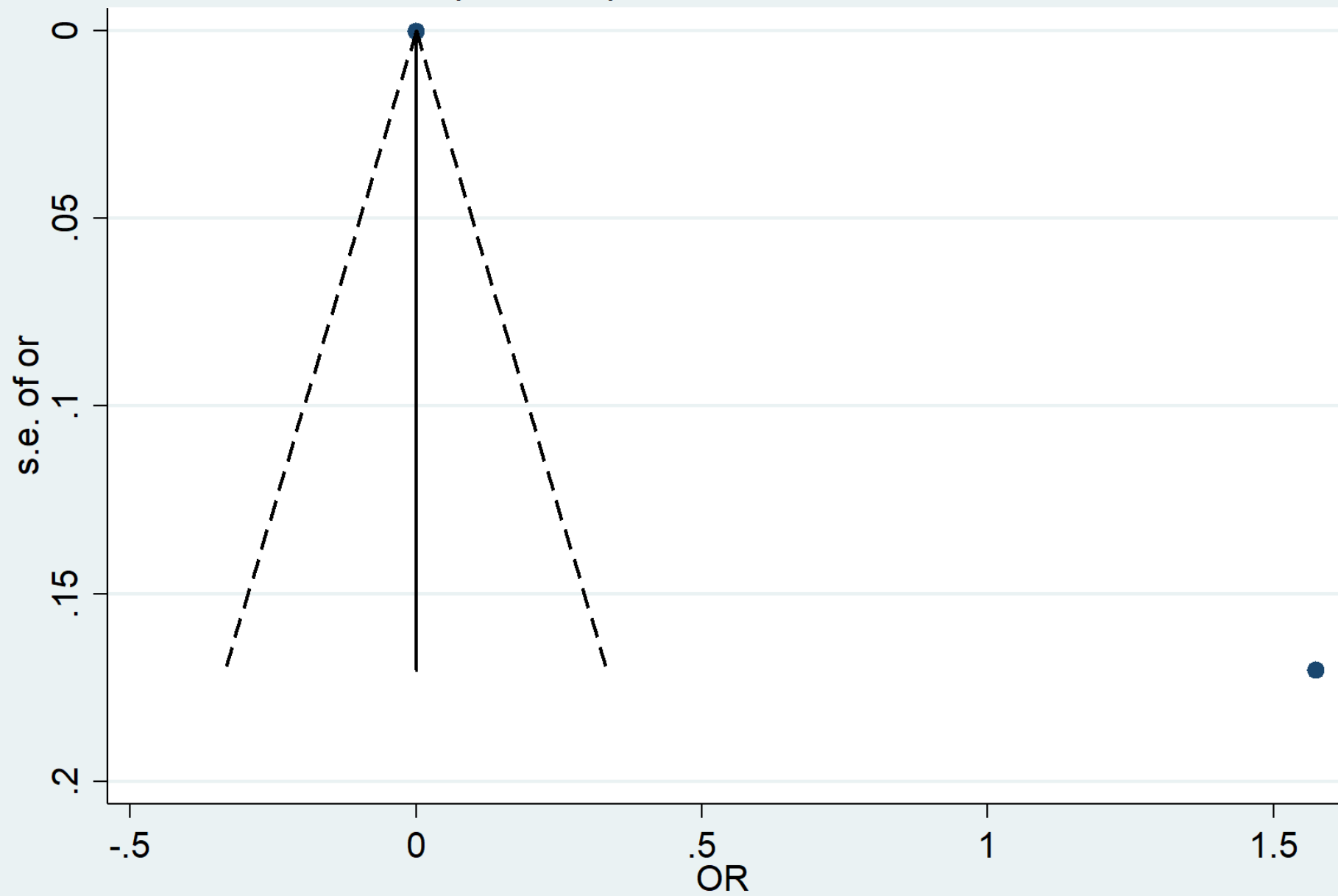

Supplement: Supplementary Figure S6 — Funnel plot of the relationship between t-IgE and response to immunotherapy in patients with allergic rhinitis. [file Datasheet6.pdf]
