# Supplementary material for: Risk factors for drug resistance in allergen immunotherapy for allergic rhinitis: a systematic review and meta-analysis
Source: Front Allergy. 2026 Jan 23;6:1743260. doi: 10.3389/falgy.2025.1743260 (PMC12876254; doi:10.3389/falgy.2025.1743260)
Supplement: Supplementary Figure S5 — Sensitivity analysis of the relationship between t-IgE and response to immunotherapy in patients with allergic rhinitis. [file Datasheet5.pdf]

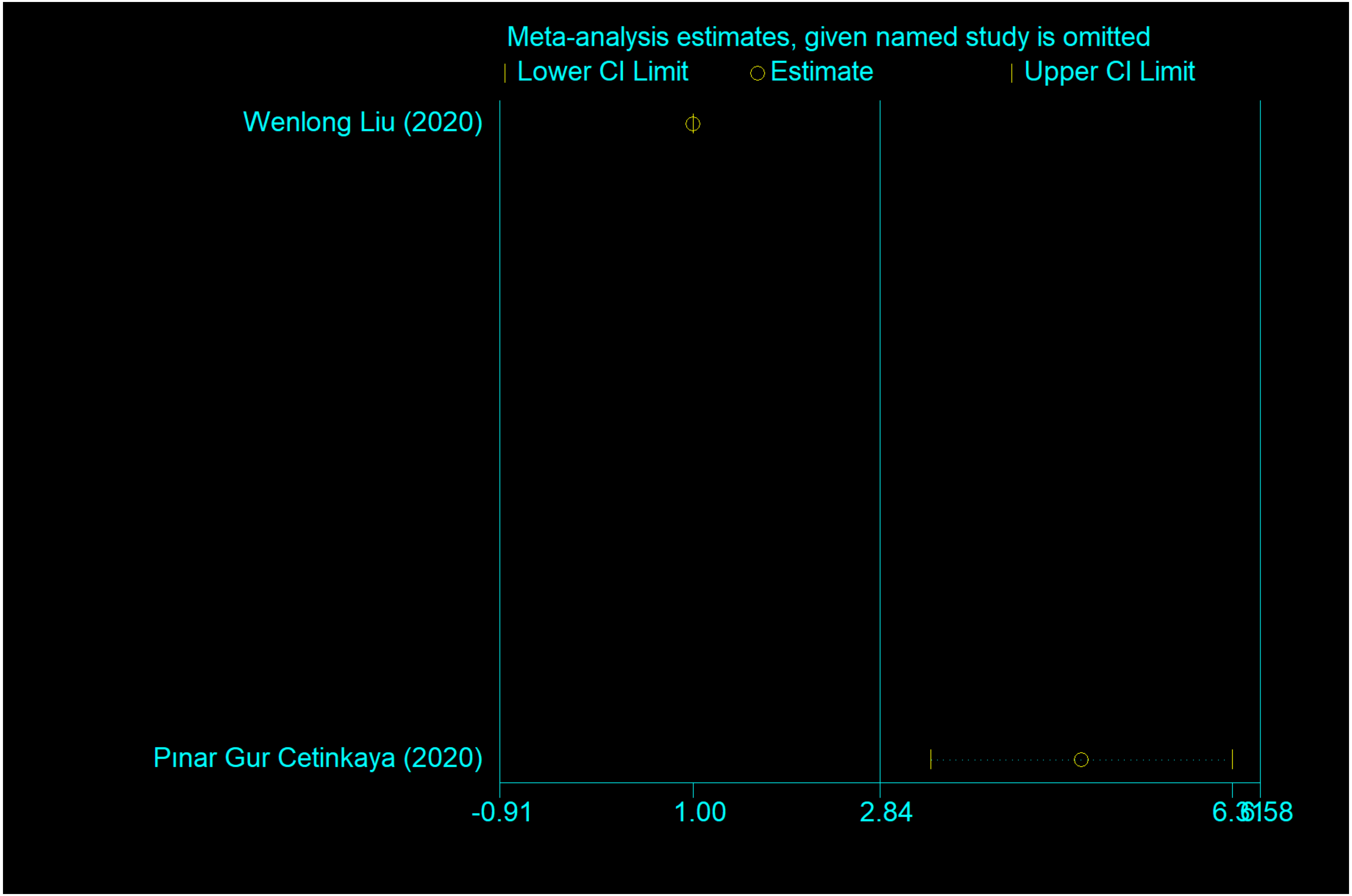
Meta-analysis estimates, given named study is omitted
Lower CI Limit
Estimate
Upper CI Limit
Wenlong Liu (2020)
Pınar Gur Cetinkaya (2020)
-0.91
1.00
2.84
6.3658
