# Supplementary material for: Risk factors for drug resistance in allergen immunotherapy for allergic rhinitis: a systematic review and meta-analysis
Source: Front Allergy. 2026 Jan 23;6:1743260. doi: 10.3389/falgy.2025.1743260 (PMC12876254; doi:10.3389/falgy.2025.1743260)
Supplement: Supplementary file 8 [file Table2.docx]

**STable 2.Publication bias test for the association between age and risk of AR resistance to AIT therapy in AR patients**

|  | | | |
| --- | --- | --- | --- |
| Regression-based Egger's test | | | |
| beta1 | 0.993 | standard error | 1.774 |
| *t* value | 0.560 | *df* value | 2 |
| *p* value | 0.632 |  |  |
| Nonparametric rank correlation (Begg) test | | | |
| Kendall’s score | 2.000 | standard error | 2.944 |
| z value | 0.679 | *p* value | 0.497 |
