# Supplementary material for: Risk factors for drug resistance in allergen immunotherapy for allergic rhinitis: a systematic review and meta-analysis
Source: Front Allergy. 2026 Jan 23;6:1743260. doi: 10.3389/falgy.2025.1743260 (PMC12876254; doi:10.3389/falgy.2025.1743260)
Supplement: Supplementary file 9 [file Table3.docx]

**STable 3.Publication bias test for the association between gender of AR patients and the risk of AR resistance to AIT treatment**

|  | | | |
| --- | --- | --- | --- |
| Regression-based Egger's test | | | |
| beta1 | -0.385 | standard error | 0.035 |
| *t* value | -11.069 | *df* value | 1 |
| *p* value | 0.057 |  |  |
| Nonparametric rank correlation (Begg) test | | | |
| Kendall’s score | -3.000 | standard error | 1.915 |
| *z* value | -1.567 | *p* value | 0.117 |
